# Outbreak of gastroenteritis highlighting the diagnostic and epidemiological challenges of enteroinvasive *Escherichia coli*, County of Halland, Sweden, November 2017

**DOI:** 10.2807/1560-7917.ES.2020.25.9.1900466

**Published:** 2020-03-05

**Authors:** Nina Lagerqvist, Emma Löf, Theresa Enkirch, Peter Nilsson, Adam Roth, Cecilia Jernberg

**Affiliations:** 1 Public Health Agency of Sweden, Solna, Sweden; 2 European Public Health Microbiology Training Programme (EUPHEM), European Centre for Disease Prevention and Control (ECDC), Stockholm, Sweden; 3 European Programme for Intervention Epidemiology Training (EPIET), European Centre for Disease Prevention and Control (ECDC), Stockholm, Sweden; 4 Clinical Microbiology, County Hospital, Halmstad, Sweden

**Keywords:** EIEC, Shigella, whole genome sequencing, food borne outbreak, ipaH

## Abstract

An outbreak of gastroenteritis with 83 cases occurred at a conference venue in November 2017 in Halland County, Sweden. Stool samples from two venue visitors and a symptomatic secondary case attributed to household transmission were PCR-positive for the *ipaH* gene, a target found in both *Shigella* spp. and enteroinvasive *Escherichia coli* (EIEC). EIEC was isolated from stool samples and whole genome sequencing analysis confirmed EIEC O96:H19 to be the aetiological agent. A cohort study was conducted among venue attendees and employees and the findings implicated contaminated leafy greens as the vehicle of infection, however, no microbiological evidence could support the study results. Here, we report the investigation into the first recorded EIEC outbreak in Sweden and illustrate the challenges associated with the differential laboratory diagnostics of *Shigella*/EIEC in an outbreak setting.

## Background

Enteroinvasive *Escherichia coli* (EIEC) and *Shigella* spp. are both Gram-negative bacteria causing diarrheal disease worldwide [1,2]. The clinical presentations of these two pathogens are very similar [3,4] and commonly manifested through diarrhoea, abdominal cramps, nausea and fever both in children and adults [5,6]. In addition to a similar clinical picture, EIEC and *Shigella* share laboratory features that can make it difficult to distinguish between them in routine clinical laboratory practice. Both pathogens are transmitted via the faecal-oral route and infections are frequently associated with consumption of contaminated food and water [7-10]. While *Shigella* is associated with large-scale food-borne outbreaks [11,12], outbreaks caused by EIEC are rarely recorded.

High prevalence of EIEC infections have been documented in rural areas and settings with poor sanitation in high-risk countries [5,13] while EIEC infections in Europe are typically sporadic and travel related [14]. Nevertheless, a few EIEC outbreaks have been reported in Europe, with the most recent ones having occurred in Italy in 2012 [15] and in the United Kingdom (UK) in 2014 [16]. These outbreaks affected 109 cases and 157 probable cases, respectively, highlighting the fact that EIEC, like *Shigella*, has the capacity to cause large gastrointestinal disease outbreaks. The outbreak strain identified in these recent European outbreaks, EIEC O96:H19, is an emergent type of EIEC that has phenotypic characteristics more resembling those of non-invasive *Escherichia coli* (*E. coli*) than those described for *Shigella* [17]. These characteristics are suggested to contribute to improved survival abilities as well as the ability to better adapt to different ecological niches [17].

Traditionally, culturing of faecal specimens has been the mainstay of laboratory diagnostics for enteric bacteria, and EIEC has been differentiated from *Shigella* by assessing a combination of several phenotypic characteristics, including biochemical, motility and serological traits [18,19]. This is now changing as PCR-based methods are becoming routine in many diagnostic laboratories [20]. In contrast to non-invasive *E. coli*, EIEC and *Shigella* can invade and multiply in intestinal epithelial cells [21], a process that is partially mediated by the products of the invasion plasmid antigen (*ipa*) genes [22]. For this reason, PCR targeting the *ipaH* gene can separate EIEC from other non-invasive *E. coli*, but cannot differentiate between EIEC and *Shigella* [23]. The *lacY* gene has been proposed as an additional molecular marker for which most *E. coli* are positive and *Shigella* is negative [24]. Its use as a PCR target in separating *Shigella* and EIEC is restricted to bacterial isolates since many faecal samples are *lacY* positive because of the presence of *E. coli* in the normal flora.

In Sweden, several clinical laboratories have shifted towards the use of direct PCR testing on faecal specimens as the primary diagnostic tool. However, most of these laboratories culture PCR-positive samples, so called PCR-guided culturing. Although culturing of PCR-positive faecal specimens is routinely performed, it can be difficult to obtain EIEC isolates since the morphology of EIEC strains on commonly used substrates can mimic the morphology of the enteric background flora, yellow colonies on xylose lysine deoxycholate (XLD) agar, rather than the morphology of *Shigella,* red colonies on XLD agar. Hence, separating EIEC from other bacteria in the normal flora usually requires additional laboratory procedures such as screening large numbers of colonies, which is considered too time consuming for most clinical laboratories. For this reason, it is likely that a patient with specimens that are *ipa*H PCR-positive but culture negative would not be notified as a case if the diagnostic algorithm at the laboratory requires a detected *Shigella* isolate. In addition, PCR is a more sensitive method than culturing [25] and *Shigella* is known for its limited survival ability in faecal samples [26], which also may lead to samples being *ipaH* PCR-positive but culture negative.

Shigellosis is notifiable by law in Sweden as in the majority of countries in Europe [27]. In 2017, the incidence was 2.1 per 100,000 inhabitants in Sweden, and the majority of cases had been infected abroad [28]. The mandatory reporting of diseases allows the implementation of a series of public health actions, including public health management and surveillance activities, and helps define risk exposures. In contrast to shigellosis, reporting is not mandatory for EIEC and the occurrence of this pathogen in Sweden is currently unknown.

## Outbreak detection

On 13 and 14 November 2017, local health authorities in Halland County and the Environmental Health Unit of Falkenberg Municipality received phone calls from three individuals who had developed gastrointestinal illness after visiting a conference and hotel venue on 8 to 10 November. The Public Health Agency of Sweden (PHAS) was notified about the suspected food-borne outbreak characterised by acute diarrhoea. Additional phone interviews with 44 individuals who had attended the venue revealed that 22 persons had experienced gastrointestinal symptoms after visiting the venue for 1 day or more during the period 8 to 10 November. An outbreak investigation team was convened with representatives from the local health authorities, PHAS, the Swedish National Food Agency and the Environmental Health Unit. The aim was to investigate the magnitude, identify the causative agent and localise the source of the outbreak.

Here, we report the findings from the outbreak investigation and illustrate the challenges associated with the differential diagnostics of EIEC and *Shigella.*

## Methods

### Epidemiological investigation

We conducted a cohort study to investigate the outbreak, and an online questionnaire was created to collect data on the dates of venue attendance, date of symptom onset, symptoms and food consumed. The food items specified in the questionnaire were obtained from the restaurant’s menu. Email addresses of venue visitors and employees were collected via contact persons for the visiting parties or the venue’s proprietors, respectively. Information on age was not collected in the questionnaire. The contacted parties visited the venue in connection to work. A link to a web-based questionnaire was sent on 17 and 20 November to 554 email addresses, and a reminder was distributed on 23 November.

### Case definition

A case was defined as an individual who consumed food and/or beverage at the conference venue during the period 8 to 10 November 2017, and reported symptoms of gastrointestinal illness including abdominal pain, nausea, diarrhoea (more than three loose stools in 24 hours), bloody diarrhoea and/or vomiting within 7 days after attending the venue, i.e. symptom onset before 15, 16 or 17 November.

### Statistical analysis

Univariate analysis of exposures was performed using the CSTABLE command [29] in Stata version 14.0 (Stata Corporation, College Station, Texas, United States (US)). We obtained attack rates (AR), risk ratios (RR) with 95% confidence intervals (95% CI) and p values (p). P values below 0.05 were considered significant. Differences of rates among the exposed and unexposed were analysed using chi-squared test.

### Microbiological investigations

The local clinical microbiological laboratory received stool samples from two outbreak cases and a suspected secondary case, and performed routine screening methods for gastrointestinal pathogens. An in-house real-time PCR approach was used targeting genes from a variety of gastrointestinal pathogens, including the *ipaH* gene. Guided by the PCR finding of the *ipaH* target, attempts were made to isolate *Shigella* from two outbreak samples by inoculation on Acumedia XLD agar (Neogen, Lansing, Michigan, US). Only yellow colonies grew on the XLD agar, indicating that no *Shigella* could grow from the specimens. Additional culturing attempts directed towards *Shigella* and EIEC were made, including dilution of samples for plate inoculation and increasing the number of colonies per sample picked and screened by PCR targeting the *ipaH* gene. Three *ipaH* PCR-positive isolates were sent to PHAS for species determination and serotyping. The isolates were phenotypically analysed using a biochemical panel of six different parameters and serotyped by agglutination using pooled antisera, all according to reference methodology [30].

### Whole genome sequencing

Bacterial DNA was extracted using the MagDEA Dx SV reagent kit and the magLEAD instrument (Precision System Science, Chiba, Japan) and sequenced on the Ion Torrent S5 XL platform (Thermo Fisher Scientific, Waltham, Massachusetts, US). Library and template preparations were performed according to the manufacturer's instructions (Thermo Fisher Scientific). The raw data was assembled to contigs using CLC assembly cell version 4.4.2 (Qiagen Bioinformatics, Hilden, Germany). Raw reads were then mapped to the assembly and the average coverage was calculated. Genomes with incorrect total size (+/− 20%) or low coverage (< 20x) were flagged as failed quality control. Multilocus sequence typing (MLST) was calculated by mapping the raw reads to a reference sequence for each loci of the Warwick *E. coli* 7-gene MLST scheme. Consensus sequences were called and compared with the allele reference databases using BLAST+ version 2.2.30. The molecular serotype was determined by comparing contigs to the SerotypeFinder version 1.0 database (Technical University of Denmark (DTU), Lyngby, Denmark) using BLAST+ version 2.2.30, the *wzx* and *wzy* genes for O-type and the *fliC* gene for H-type. Single nt polymorphisms (SNPs) were called based on an assembly of one of the outbreak strains using CLC assembly cell (minimum 10x coverage, 90% read consensus). Minimum spanning trees were generated using MSTgold [31] and recombinations were filtered by looking for SNPs with a pairwise distance of 500 nt. The sequences were deposited in the European Nt Archive (ENA) under accession numbers ERS3907567, ERS3907568 and ERS3907569.

### Environmental investigations

On 15 November, environmental health officers inspected the restaurant at the venue and reviewed its routines for food handling, storage and kitchen hygiene, as well as collected leftover food samples that had not been consumed or thrown away. Sampled food items were cooked duck, mousse, blueberry meringue, vanilla pastry, pickled red cabbage, bleak roe and freeze-dried blueberries. The food samples were tested for *E. coli* at commercial laboratories. Leftover frozen dill was later collected and tested for *E. coli* at the Swedish National Food Agency.

### Ethical statement

Written consent was obtained from all participants who provided questionnaire answers. Ethical approval was not required as the investigation was performed under a mandate of The Public Health Agency of Sweden in its remit to undertake outbreak investigations regarding national communicable disease control in the interest of public health.

## Results

### Descriptive analysis

From the list of 554 eligible individuals who received the web-based questionnaire, 351 visitors and 47 staff members completed the survey (response rate: 72%). Of these, 57% (n = 228) were female and three did not provide information on sex. The case definition was met by 83 individuals (78 visitors and 5 staff members), of whom 67% (n = 56) were female and one did not provide information on sex. The overall attack rate was 21% (83/398), and was higher for women (25%; 56/228) than men (16%; 26/167). The date of onset of symptoms ranged from 8 to 14 November, with the peak occurring on 10 and 11 November (Figure). Three cases did not provide information on symptom onset. Fourteen cases reported their symptoms to last for 1 day or less, 11 cases each reported symptom duration of 2 or 3 days, five cases reported 4 days, but a majority of the cases (n = 42; 51%) were symptomatic for 5 or more days. Symptoms reported by the cases were abdominal pain (n = 74; 89%), bloody diarrhoea (n = 4; 4.8%), diarrhoea (n = 73; 88%), nausea (n = 55; 66%) and vomiting (n = 8; 9.6%).

**Figure:**
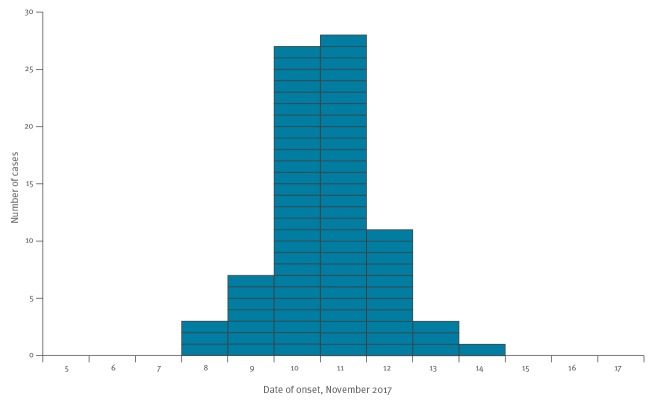
Onset of gastroenteritis symptoms of outbreak cases among conference and hotel venue attendees and personnel, County of Halland, Sweden, November 2017 (n = 83)

### Food exposure

The venue served breakfast, lunch, dinner and snacks during coffee breaks to hotel and conference attendants and staff members, and was open to the public for lunch and dinner servings. An estimated 1,000 portions were served between 8 and 10 November. Of the 398 respondents, 173 individuals including 32 cases attended the venue on 8 November (RR: 0.82; 95% CI:0.55–1.21; p = 0.310), 341 individuals including 80 cases attended the venue on 9 November (RR: 4.46; 95% CI: 1.46–13.63; p = 0.002), and 129 individuals including 35 cases attended the venue 10 November (RR: 1.52; 95% CI: 1.04–2.23; p = 0.033). None of the 41 individuals who solely visited the venue on 8 November experienced gastrointestinal symptoms and exposure to meals served on 8 November were not significantly associated with illness (Table 1). The highest risk ratios were observed for attending meals served between the morning and afternoon coffee breaks on 9 November, and food consumption at lunch (Table 1). Food items served on 9 and 10 November were analysed for association with illness. Food items significantly associated with disease were served during lunch on 9 November and are shown in Table 2. No strong association was observed between illness and consumption of a specific food item (Table 2). The ambiguous results led to additional interviews with kitchen staff, who informed the outbreak investigation team that a variety of leafy green products, including baby spinach, rocket salad and mixed salads, were used as garnish on or in dishes (previously undisclosed ingredients).

**Table 1:** Attack rate and crude risk ratios for gastroenteritis among conference and hotel venue attendees and personnel, by meals, 8–10 November 2017, County of Halland, Sweden (n = 398 survey respondents)

Date	Exposure	Exposed			Non-exposed			RR	95% CI	p value
		Cases (n)	Total (N)	AR (%)	Cases (n)	Total (N)	AR (%)			
8 Nov	Breakfast	11	63	18	72	335	22	0.81	0.46–1.44	0.470
	Coffee break	17	70	24	66	328	20	1.21	0.76–1.92	0.436
	Lunch	24	147	16	59	251	24	0.69	0.45–1.07	0.089
	Afternoon coffee	22	110	20	61	288	21	0.94	0.61–1.46	0.795
	Dinner	26	103	25	57	295	19	1.31	0.87–1.96	0.203
9 Nov	Breakfast	32	127	25	51	271	19	1.34	0.91–1.97	0.144
	Coffee break	65	244	27	18	154	12	2.28	1.41–3.69	0.000
	Lunch	75	301	25	8	97	8	3.02	1.51–6.04	0.000
	Afternoon coffee	54	192	28	29	206	14	2.00	1.33–3.00	0.001
	Dinner	27	93	29	56	305	18	1.58	1.06–2.35	0.027
10 Nov	Breakfast	27	80	34	56	318	18	1.92	1.30–2.83	0.001
	Coffee break	24	68	35	59	330	18	1.97	1.33–2.93	0.001
	Lunch	31	98	32	52	300	17	1.82	1.25–2.67	0.002
	Afternoon coffee	8	25	36	74	373	20	1.81	1.04–3.18	0.054
	Dinner	6	23	26	77	375	21	1.27	0.62–2.60	0.525

AR: attack rate; CI: confidence interval; RR: risk ratio.

**Table 2:** Attack rate and crude risk ratios for gastroenteritis among conference and hotel venue attendees and personnel, by food item served at lunch, 9 November 2017, County of Halland, Sweden (n = 301 survey respondents)

Exposure	Exposed			Unexposed			RR	95% CI	p value
	Cases (n)	Total (N)	AR (%)	Cases (n)	Total (N)	AR (%)			
Root vegetables (salad buffet)	50	152	33	25	149	17	1.96	1.28–2.99	0.001
Beans (salad buffet)	30	78	38	45	223	20	1.91	1.30–2.80	0.001
Plaice	51	160	32	24	141	17	1.87	1.22–2.88	0.003
Julienne vegetables^a^	29	81	36	28	140	20	1.79	1.15–2.78	0.010
Raw vegetables (salad buffet)	26	70	37	49	231	21	1.75	1.18–2.59	0.007
Tomato and onion salad (salad buffet)	48	152	32	27	149	18	1.74	1.15–2.64	0.007
Bean salad	21	55	38	54	246	22	1.74	1.15–2.62	0.012
Leafy greens (salad buffet)	45	152	30	30	149	20	1.47	0.98–2.20	0.058

AR: attack rate; CI: confidence interval; RR: risk ratio.

^a^ n = 221

### Microbiological investigations

Stool samples from three individuals, two venue visitor cases and a secondary case attributed to household transmission, tested positive for the *ipaH*-gene and were PCR-negative for other common gastrointestinal pathogens. Isolates were successfully retrieved from these three stool samples by culturing at the local laboratory and were sent to the reference laboratory for further characterisation. The outbreak strain was positive for β-galactosidase and lysine decarboxylase activity (LDC), fermented sugars such as glucose and mannose, was motile, and tested negative in *Shigella* agglutination tests. For this reason, the isolates were defined as EIEC.

Data obtained from whole genome sequencing (WGS) was used to determine the molecular serotype, the sequence type (ST) and to assess genetic similarity among the isolates. The isolates were *E. coli* serotype O96:H19, a known EIEC serotype, and were classified as ST99. The three isolates showed high genomic sequence similarity (0–1 SNP difference, 99.8% of the genome). Isolates collected from the two venue visitors showed indistinguishable genomes (no SNP difference) while the genome of the isolate collected from the secondary case differed from the other two in one SNP. We compared the genomic sequences obtained during this outbreak to published sequences [17] and the SNP-based phylogeny showed large genomic differences (> 100 SNPs) between this outbreak strain and previously published EIEC O96:H19 outbreak strains (data not shown).

None of the food items sent for testing at the commercial laboratories or the frozen dill tested at the Swedish National Food Agency tested positive for *E.coli*.

## Outbreak control measures

Representatives from the Environmental Health Unit performed a post-outbreak inspection of the kitchen and had no remarks. Officers performing routine inspections earlier that year came to the same conclusion. Perishable food items served between 8 and 10 November were already consumed or had been discarded when the outbreak control team arrived. Food inspectors traced back suspected food items including baby spinach, rocket salad and mixed leafy greens (including rocket salad, baby spinach and red leafy greens). Trace-back investigations identified a distributor who delivered these items to the venue on 7 November (baby spinach and mixed leafy greens), 8 November (rocket salad and mixed leafy greens) and 9 November (baby spinach, rocket salad and mixed leafy greens). The food items were received from a producer who trades with leafy greens and packages pre-washed, ready-to-eat leafy greens in bags, with products delivered 7 to 9 November originating from Italy and Sweden. The producer reported that they did not have any indication from their own microbiological testing of contaminated leafy greens during the reported dates and they had not received any complaints from other buyers. For these reasons, further control measures were not undertaken.

## Discussion

This outbreak, with 83 self-reported cases and one secondary case attributed to household transmission, is the first reported EIEC outbreak in Sweden. The result of the outbreak investigation was inconclusive and no food item could be directly linked to the outbreak. However, leafy greens were suspected to be the vehicle of infection as this food item was present in a number of dishes associated with disease. The attack rate (21%) was high, also suggesting that the causative agent may have been present in many of the dishes. There was no microbiological evidence to identify the source or vehicle of infection, however, consumption of contaminated salad greens have been associated with previous EIEC outbreaks [15,16]. The trace-back of the leafy greens did not implicate that these products were contaminated before arriving at the venue. However, this scenario cannot be excluded since several reports have shown that many outbreaks involving fresh produce are linked to contamination at the field level [32].

EIEC outbreaks have shown to affect individuals of all ages [15,16]. A weakness of our study is that we did not collect information on age in the online questionnaire. However, as we only received contact information to venue employees or individuals visiting the venue in connection to work, all individuals were of working age.

In an outbreak reported in the UK in 2014, most of the food handlers who tested positive for EIEC were asymptomatic carriers and investigators identified inadequate handwashing facilities and food handling practices at the restaurant [16]. In the current outbreak, all five staff members that met the case definition had symptom onset at least 1 day after the first reported case among venue visitors. A limitation to our study is that we did not perform any interviews with kitchen staff regarding previous disease and travel history, and no members of staff were tested for asymptomatic EIEC carriage. The reason for this was that the outbreak ended quickly, suggesting that the source had been removed, and the officers inspecting the kitchen had no remarks on the facilities or the practices employed by the kitchen staff.

Despite the prompt notification to the reference laboratory and the efforts taken by the local clinical laboratory to retrieve isolates, i.e. implementing procedures to isolate EIEC to support the outbreak investigation, and obtaining the results from the WGS analysis, the definite result from the microbiological investigation was not available until 2 to 3 weeks after the outbreak was resolved. In Sweden, few laboratories actively search for EIEC in samples PCR-positive for *ipaH* and the probability of isolating EIEC during attempts to isolate *Shigella* is low. Further, culturing of *Shigella* may fail because of the pathogen’s limited survival ability in faecal samples [26]. For this reason, a number of *ipaH*-positive samples will likely be culture-negative even if routines to isolate EIEC are implemented. As culture-independent diagnostics, i.e. PCR diagnostic testing, is becoming the method of choice at primary clinical laboratories, members of an outbreak investigation team need to be aware of the limitations of an *ipaH* PCR-positive finding and that a definite microbiological confirmation can take time, or will never be achieved.

This outbreak was identified because of the high number of persons falling ill during a short time period and because they all could quickly be linked to the same venue. Only two cases and one household contact were laboratory-confirmed. This can possibly be explained by several factors, including mild cases not seeking medical care, samples not being taken in patients presenting with short-term gastrointestinal symptoms or cases not being notified in the official reporting system because samples were only *ipaH* positive.

Despite the limited number of laboratory confirmed cases, we concluded EIEC to be the disease-causing pathogen. This is based on the absence of other common gastrointestinal pathogens in the collected stool specimens and the results of the WGS analysis revealing almost identical genomic sequences of the three EIEC isolates. WGS is becoming the routine analysis tool for typing at many public health reference laboratories [33]. The continuously increased use of WGS does not only allow identification of clustering among outbreak cases as shown in our study, but also provides knowledge on the genetic distribution of EIEC. This improves our understanding of what specific serotypes and additional marker genes are associated with EIEC, possibly enabling better molecular differentiation of *Shigella* and EIEC in the future.

In Sweden, the occurrence of EIEC is currently unknown and no information on the distribution of the specific outbreak strain was available to the outbreak investigation team, i.e. it is unknown if EIEC O96:H19 is circulating in Sweden or if this strain was introduced via an imported food item. This specific EIEC serotype of ST99 was first reported as the disease-causing pathogen in an outbreak in Italy in 2012, and has since been implicated in two outbreaks in the UK and a sporadic travel-related case in Spain [17]. The O96:H19 serotype of ST99 is considered a new emerging virulent EIEC strain [19] and differs from other traditional EIEC and *Shigella* strains in many phenotypic tests as it is more reactive, e.g. ferments glucose, is positive in LDC and is motile [17]. This was also shown in the present investigation. Emerging EIEC strains such as EIEC O96:H19, which phenotypically resembles *E. coli* more than *Shigella* which could enable improved survival abilities [17], could potentially contribute to an increase in food-borne outbreaks caused by EIEC in the future. This necessitates improved laboratory preparedness and consensus on recommendations for public health measures of PCR-positive *Shigella*/EIEC faecal samples.
